# A scheduling route planning algorithm based on the dynamic genetic algorithm with ant colony binary iterative optimization for unmanned aerial vehicle spraying in multiple tea fields

**DOI:** 10.3389/fpls.2022.998962

**Published:** 2022-09-16

**Authors:** Yangyang Liu, Pengyang Zhang, Yu Ru, Delin Wu, Shunli Wang, Niuniu Yin, Fansheng Meng, Zhongcheng Liu

**Affiliations:** ^1^School of Engineering, Anhui Agricultural University, Hefei, China; ^2^School of Mechanical and Electronic Engineering, Nanjing Forestry University, Nanjing, China; ^3^School of Mechanical Engineering, Yangzhou University, Yangzhou, China

**Keywords:** multi-tea field plant protection, unmanned aerial vehicle, hilly mountainous area, bionic algorithm, scheduling route planning

## Abstract

The complex environments and weak infrastructure constructions of hilly mountainous areas complicate the effective path planning for plant protection operations. Therefore, with the aim of improving the current status of complicated tea plant protections in hills and slopes, an unmanned aerial vehicle (UAV) multi-tea field plant protection route planning algorithm is developed in this paper and integrated with a full-coverage spraying route method for a single region. By optimizing the crossover and mutation operators of the genetic algorithm (GA), the crossover and mutation probabilities are automatically adjusted with the individual fitness and a dynamic genetic algorithm (DGA) is proposed. The iteration period and reinforcement concepts are then introduced in the pheromone update rule of the ant colony optimization (ACO) to improve the convergence accuracy and global optimization capability, and an ant colony binary iteration optimization (ACBIO) is proposed. Serial fusion is subsequently employed on the two algorithms to optimize the route planning for multi-regional operations. Simulation tests reveal that the dynamic genetic algorithm with ant colony binary iterative optimization (DGA-ACBIO) proposed in this study shortens the optimal flight range by 715.8 m, 428.3 m, 589 m, and 287.6 m compared to the dynamic genetic algorithm, ant colony binary iterative algorithm, artificial fish swarm algorithm (AFSA) and particle swarm optimization (PSO), respectively, for multiple tea field scheduling route planning. Moreover, the search time is reduced by more than half compared to other bionic algorithms. The proposed algorithm maintains advantages in performance and stability when solving standard traveling salesman problems with more complex objectives, as well as the planning accuracy and search speed. In this paper, the research on the planning algorithm of plant protection route for multi-tea field scheduling helps to shorten the inter-regional scheduling range and thus reduces the cost of plant protection.

## Introduction

Longjing tea is known for its unique fragrance and high quality, is grown exclusively in many tea areas, including the West Lake mountainous area of Hangzhou City, Zhejiang Province, the Lion Peak Mountain. This region is known for producing top Longjing varieties of tea Lion Peak Longjing, which contains more amino acids, catechins, chlorophyll, vitamin C and other components compared to other tea varieties, and has important nutritional and economic value. Effective fertilization and irrigation applications can guarantee the yield and quality of tea in daily tea garden management (Xia et al., 2019; Chen, 2021). However, tea gardens are generally planted in mountainous and hilly terrain, and thus the tea field is small and scattered, making it difficult for plant protection machinery to enter the area. Furthermore, the nutrient loss during the manual protection of plants is serious, and the yield and quality of the tea are difficult to guarantee. Unmanned aerial vehicle (UAV) plant protection procedures are simple to operate, have a high work efficiency, and are unaffected by terrain and crop growth. Such techniques are widely adopted in agricultural surveying and mapping, plant protection (Ali et al., 2017; Zhang et al., 2019; Li F. et al., 2020; Asadzadeh et al., 2022). For plant protection applications, the UAVs typically take-off and land vertically in the tea garden. The high-speed downward swirling airflow, and water and fertilizer spraying devices are able to turn the tea leaves, allowing for the even spraying of the front and back of the leaves (Lan et al., 2010; Kose and Oktay, 2020; Qin et al., 2021; Wang et al., 2021; Zhan et al., 2021; Zhu et al., 2021; Şahin et al., 2022). UAV multi-tea field scheduling route planning is an important component of the precision planting and protection of tea fields. However, it can prove to be technically complicated for UAV planting and protection applications and restricts the development of aerial precision operations in hilly mountainous areas (Lan and Chen, 2018; Hu et al., 2021).

The development of intelligent algorithms has resulted in further advancements in the route planning problem. Jiang et al. (2021) designed a UAV route planning algorithm based on the segmented fitness strategy by adding constraints to the fitness function of the algorithm, thus avoiding the premature convergence defect and adapting to the route planning of more complex environments. Wu et al. (2019) proposed a path planning method based on the beetle search algorithm, overcoming the trade-off between high computational complexity and the UAV requirement for real-time trajectory planning. The offline UAV path planning method based on the improved particle swarm algorithm proposed by Huang (2021) can realize the planning of 3D routes. This algorithm greatly reduces the computational effort and improves the route planning efficiency. Qu et al. (2020) proposed a UAV path planning method with a hybrid gray wolf optimization algorithm, which combines the gray wolf optimization algorithm and the symbiotic biological search method to smooth the generated routes and make the routes more suitable for UAVs. However, these aforementioned studies are limited to planning of UAV remote sensing and obstacle avoidance routes (Wu et al., 2019; Qu et al., 2020; Huang, 2021; Jiang et al., 2021), which is different from the scheduling problem and cannot be applied to the UAV multi-tea field scheduling route planning scenario. Pang et al. (2021) proposed an adaptive route planning approach based on an artificial potential field method. Moreover, Shao et al. (2019) developed a multi-helicopter search and rescue route planning strategy based on the optimization strategy algorithm, while a helicopter scheduling route planning algorithm based on the operational area entry point mechanism was also proposed by the group Fang et al. (2021). These algorithms can be applied for the planning of helicopter scheduling routes and shorten the scheduling operation distance between regions. However, those presented in above are restricted to the route planning of manned aircrafts (Shao et al., 2019; Fang et al., 2021; Pang et al., 2021), and are thus unable to meet the operating accuracy of UAVs. There is currently a lack of research on the optimal scheduling route planning for UAV spraying in multi-tea fields.

Scholars across the globe have conducted some research on the UAV route planning problem, resulting in various route planning algorithms. For example, Li K. et al. (2020) proposed an improved fruit fly optimization algorithm based on the optimal reference point to study the problem of UAV task assignment with task priority and changeable tasks. The method is able to achieve the optimal initial trajectory for multiple UAVs. Yan et al. (2021) developed a multi-UAV task allocation algorithm based on an improved particle swarm optimization algorithm, this algorithm improves the traditional particle swarm algorithm by introducing partial matching crossover and secondary transposition mutation to effectively improve the efficiency of UAV task assignments in marine environment and can optimize the navigation path. Tang et al. (2021) proposed a joint global and local path planning optimization for UAV task scheduling towards crowd air monitoring, which achieves the effective utilization of UAV airborne resources by improving the mutation mechanism and adaptive inertia weights of the particle swarm algorithm. Liu et al. (2021) proposed an optimization method based on the divide and conquer framework for the integrated scheduling of multiple UAVs, which divides the task scheduling problem of multiple UAVs into a task allocation phase and a single UAV scheduling phase, and is able to achieve task allocation among multiple UAVs. Wang et al. (2020) proposed a XGBoost-enhanced fast constructive algorithm, and an embedded insertion-based heuristic algorithms with different sequencing rules, then, through experiments on the Meituan delivery platform dataset, it is verified that the method can obtain shorter UAV delivery paths and save some computation time. Shafiq et al. (2021) designed a UAV path planning scheme that combines a Maximum-Minimum ant colony optimization with a vicsek based multi-agent system, which overcomes the shortcomings of traditional path planning algorithms in terms of controlling and synchronizing information globally. Xu et al. (2015) designed a UAV planning algorithm with minimal energy consumption by dividing the plant protection area through the grid method and reasonably allocating the spraying volume and return points of each sortie, which minimizes the total energy consumption of the UAV’s work, reduces the invalid consumption of energy by the UAV in non-operating situations, and improves the UAV’s operating efficiency. Although all of the aforementioned algorithms can achieve the shortest flight range for their related problems (Xu et al., 2015; Li K. et al., 2020; Wang et al., 2020; Liu et al., 2021; Shafiq et al., 2021; Tang et al., 2021; Yan et al., 2021), they differ from the multi-tea field planting route scheduling planning problem in the following three aspects: (i) the types of problems solved by each study are different, such as the vehicle routing problem, the quadratic assignment problem, the traveling salesman problem, etc.; (ii) the applied UAV sorties and models are different, in particular, this paper focuses on the scheduling route planning problem for the single sortie of plant protection UAVs; and (iii) the environment and work content of the application are different to those of the current literature. At present, there are few studies on the planning of plant protection routes for UAVs in hilly and mountainous areas. Therefore, this paper proposes a method that can realize single-sortie multi-tea field plant protection scheduling route planning.

The structure of this paper is shown as below. Section 2 describes in detail the specific method of full-coverage spraying routes in the region and the specifics of improving DGA-ACBIO, including dynamic crossover strategy, dynamic variation strategy, the concept of iterative cycles, and binary iterative pheromone update strategy; Section 3 conducts comparison experiments on the multiple tea fields problem and standard traveling salesman problems, and shows the results of the significant level difference between the algorithm in this paper and other algorithm; Section 4 provides a detailed analysis of the comparative experimental results of each algorithm; Finally, Section 5 concludes with an integrated summary and prospect of this paper.

In order to achieve the fast and efficient planning of multi-tea field plant protection routes and to shorten the scheduling range between fields, this study proposes a serial fusion scheduling route planning algorithm that combines the adaptive dynamic genetic algorithm with the ant colony binary iterative algorithm by designing the underlying logic of the fusion algorithm. The algorithm can effectively improve the search accuracy, convergence speed and stability, and achieve the purpose of quickly planning the optimal scheduling route for UAV multi-tea field plant protection. The algorithm can streamline the route, improve the planting efficiency, improve the planting effect, and thus improve the yield and quality of tea. The results can provide basic theoretical support for the research of aerial precision operation technology for multi-tea fields in hilly mountainous areas.

## Details of optimization techniques

### Environmental modeling of tea fields

**FIGURE 1:**
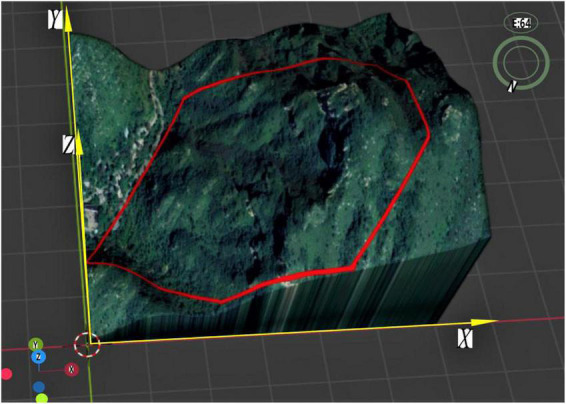
Projection coordinates of a single tea field.

The correct flight heading must be maintained during UAV operations, and thus the terrain should be perfectly reproduced on the aerial map during the environmental modeling. The map projection “Mercator projection” ensures that there is no angular distortion following the projection (Wada, 2019), and is thus selected for the aerial map to minimize the flight offset. In order to facilitate the analysis, the operation area is projected into the first quadrant of the coordinate system in this study (Figure 1). It is assumed that the application area is an arbitrary n-sided polygon denoted as P1P2……Pn, and the point with the lowest latitude and longitude in the operation area is set as the coordinate origin of the projection plane. Environmental coordinate system Z is established by setting the due east direction, due north direction and altitude as the positive direction of the x-axis, the positive direction of the y-axis, and the positive direction of the z-axis, respectively (Figure 1).

By taking the efficiency of DJI plant protection UAV T20 (12 hm^2^/h) as a reference (DJI-Innovations, 2019), we modeled a tea field near Lion Peak Mountain in the Xihu Mountains of Hangzhou, Zhejiang Province, China. A total of 20 tea fields with a mean value of 0.45 hm^2^ were selected. The length and width of the tea field were set as [0,650] and [0,550], respectively, in order to ensure that the plant protection UAV could complete the plant protection operation in at least 1 h. Ovital 3D (V9.1.6 X64) was employed to establish the tea field model. The latitude and longitude coordinates of each tea field vertex are shown in Supplementary Table 1.

**FIGURE 2:**
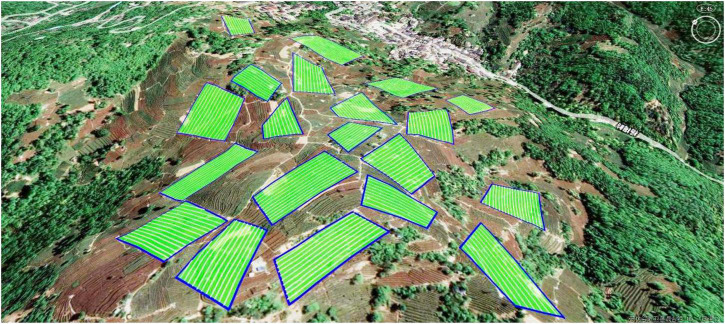
Multi-tea field model.

The single tea field modeling method was used to model the tea gardens of the 20 tea fields. Figure 2 depicts the model, where the blue solid line and green area denote the boundary and area of each tea field, respectively.

### Single tea area full coverage spraying route planning

UAV plant protection routes are divided into regional full coverage and inter-regional scheduling routes. Full coverage route planning denotes the planning of the shortest route covering all areas within a pre-defined space according to the width of the spray pattern.

A UAV using variable application technology can adjust spray volume based on the height and flight speed and ensure a consistent canopy application across tea trees. This technology simplifies the application process from multiple low-slope tea areas into a single plane application. On this basis, to ensure full coverage when spraying, the spraying route needs to extend beyond the boundaries of the operating area. Therefore, spraying outside the designated area is minimized, and the spraying range is more precise if the length of the spraying route outside the boundary is reduced. At a given spray width, when the number of turnarounds is low, the individual routes are longer. Such routes are more suitable for UAVs. The method employed to determine the suitable spraying route for a single tea field is described below.

**FIGURE 3:**
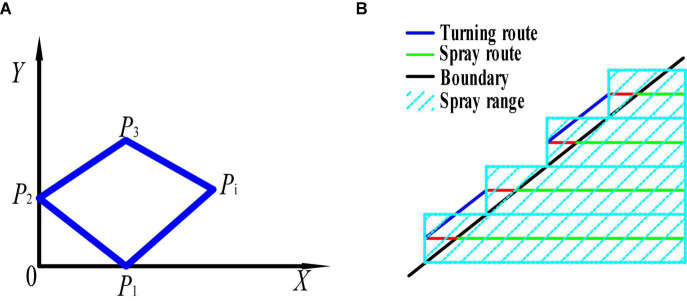
A diagram of the spraying area and the spraying route in the border area of the single tea field. **(A)** Spray area; **(B)** the route to the border of the tea field.

Establish a coordinate system for the operating area of the tea area. It is assumed that the operation area is a polygon with n sides and is located in the eastern longitude and northern latitude region. For the convenience of analysis, the polygonal application operation area was located in the first quadrant of the coordinate system. Furthermore, the smallest latitude and longitude values in the operation area are selected as the coordinate origin. The positive direction of the x-axis is due east and the positive direction of the y-axis is due north to establish the environmental coordinate system (Figure 3A).

(1) Calculate the slope of each tea field boundary as follows.


(1) $$\left\{ \begin{array}{c} k_{j}=\frac{\left|y_{j+1}-y_{j}\right|}{\left|x_{j+1}-x_{j}\right|},1\leq j<n,j\in Z\\ k_{j}=\frac{\left|y_{1}-y_{j}\right|}{\left|x_{1}-x_{j}\right|},j=n,j\in Z \end{array}\right.$$

where *k*_*j*_ is the slope of the jth edge; (*x*_*j*_,*y*_*j*_) is the coordinate of the *j*th vertex; *Z* is an integer.

(2) Calculate the total spray range outside the application operation area. Since most aerial applications use parallel flight paths, the x-axis is selected as the starting edge and the number of paths is calculated using Equation (2) to achieve full coverage.


(2) $$\left\{ \begin{array}{c} M_{j}=\frac{\left|y_{j+1}-y_{j}\right|}{W_{a}},1\leq j\,n,j\in Z\\ M_{j}=\frac{\left|y_{1}-y_{j}\right|}{W_{a}},j=n,j\in Z \end{array}\right.$$

(3) $$M=\left\lceil \frac{1}{2}\,\sum_{j=1}^{n}M_{j}\right\rceil$$

where *M*_j_ is the number of application paths required for the jth edge. *M* is the total number of application paths. *W*_*a*_ is the spray width.

Then, the spray range outside the application area is then calculated. Since the spray routes are parallel and the spacing is the spray range, the quadrilateral ABCD shown in Figure 3B is a parallelogram. Therefore, it is obtained that.


(4) $$r_{A\,B}=\frac{W_{a}}{2\,k}$$

where *r*_*AB*_ is the range of each spray route outside the operating area, and *k* is the slope of the boundary line of the operating operation area.

Since the total out-of-area spray range on each boundary is equal to the sum of each out-of-area range from that boundary line, combining Equations (2) and (4) gives the out-of-area spray range on each boundary.


(5) $$r_{j}=\frac{M_{j}\,W_{a}}{2\,k_{j}}$$

where *r*_*j*_ is the out-of-area spray range at the jth boundary. Equations (1) and (5) are combined to obtain Equation (6) as follows.


(6) $$\left\{ \begin{array}{c} r_{j}=\frac{\left|x_{j+1}-x_{j}\right|}{2},1\leq j<n,j\in Z\\ r_{j}=\frac{\left|x_{1}-x_{j}\right|}{2},j=n,j\in Z \end{array}\right.$$

The total spray range outside the area of application is equal to the sum of all spray ranges, i.e.


(7) $$R=\frac{\left|x_{1}-x_{n}\right|}{2}+\sum_{j=2}^{n-1}\frac{\left|x_{j+1}-x_{j}\right|}{2},j\in Z$$

where *R* is the total spray range outside the application area.

As shown in Equation (7), when the spray route is parallel to the x-axis, and the x-axis of the established coordinate system is parallel to the boundary of the application operation area, the shortest total spray range outside the area can be obtained. That is, the spray range when the spray route is parallel to the boundary line of the area is shorter than the spray range when it is not parallel to the boundary.

Determine the coordinate system of the shortest spraying range. This is done by taking each boundary of the application area as the x-axis and establishing the coordinate systems *Z*_1_, *Z*_2_ and *Z*_*n*_ with the starting endpoint of each boundary as the coordinate origin, then calculated and compared using Equation (7), resulting in the shortest total spray range *R*_*i*_ outside the operating area for n coordinate systems, at which point the coordinate system *Z*_*i*_ is the shortest spray range coordinate system.

When the total spray range outside the application operation area is 0 in all coordinate systems i.e., the application operation area is a rectangular area it is necessary to further compare the size of the spray areas *S*_*b*_ and *S*_*a*_ outside the area. If *S*_*b*_ (*S*_*a*_) is smaller, the coordinate system established with the width (length) of the rectangle as the x-axis is the shortest spray range coordinate system.


(8) $$S_{b}=\left\lceil \frac{r_{a}}{W_{a}}\right\rceil \,W_{a}\,r_{b}-S_{0}$$

(9) $$S_{a}=\left\lceil \frac{r_{b}}{W_{a}}\right\rceil \,W_{a}\,r_{a}-S_{0}$$

where *S*_*b*_ is the spraying area outside the area when the spraying route is parallel to the width of the rectangle; *S*_*a*_ is the spraying area outside the area when the spraying route is parallel to the length of the rectangle; *r*_*a*_ is the length of the rectangle; *r*_*a*_ is the width of the rectangle; *S*_0_ is the area of the application operation area.

**FIGURE 4:**
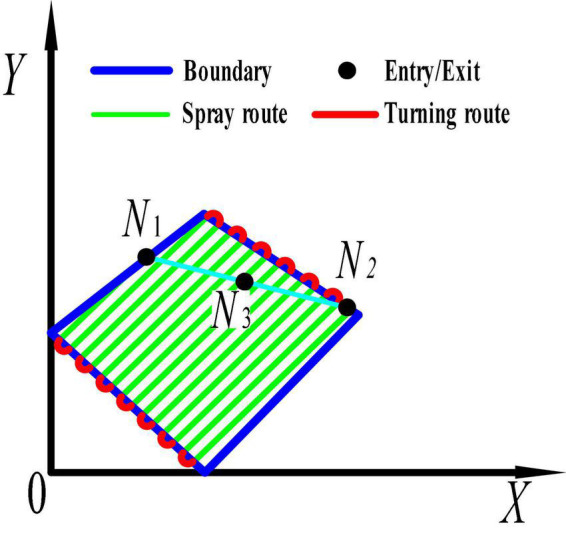
acquisition of vertices in tea field.

(4) The full-coverage route of a single tea field is planned by the full-coverage planning method proposed in this paper. That is, under the coordinate system *Z*_*i*_, the full-coverage route planned by making the spray route parallel to the x-axis is the route with the shortest spraying range and the smallest excess coverage, as shown in Figure 4. Then, previous research of our group on a regional full-coverage route planning algorithm demonstrated that in each operation area, there is only one optimal full coverage route planned (Liu et al., 2020), that is, there is only one start and end point for the route. We select midpoint *N*_3_, starting point *N*_2_ and ending point *N*_1_ of the target area as the characteristic points representing the area. This transforms the multi-regional scheduling route planning problem into a traveling salesman problem (Figure 4).

### Design of route planning algorithm for multi tea field scheduling

#### Permission to reuse and copyright

Prior to the running of the genetic algorithm, we employ binary coding to encode X work areas, with each segment corresponding to a work area node (Roberge et al., 2013), where integer X*∈ 0,1…,7. This indicates that there are eight work areas, with the arrangement of randomly generated chromosome codes set as 010| 110| 100| 101| 111| 011| 001| 000|. The fitness function indicates the individual’s ability to adapt to the environment; the larger the fitness function value, the stronger the individual’s adaptability, that is, the better the route. We set | *k*_1_| *k*_2_| … | *k*_*i*_| … | *k*_*n*_| as the coded chromosome, with a fitness function described as in Equation (10):


(10) $$f_{n}=\frac{1}{\sum_{i\,1}^{n}D_{k_{i}\,k_{j}}}$$

where *D*_*kikj*_ is the distance from operating area i to operating area *j*; and *f*_*n*_ is the fitness, defined as the reciprocal of the distance required to return to the starting node after traversing all nodes.

(1) Crossover operator optimization;

In order to enable the algorithm to maintain a high search efficiency at all stages of the population evolution, we account for the crossover probability and set the value between [0.6,1.0] (Wang and Han, 2021). Here, we propose a method for dynamic crossover probability $P_{c}^{\prime }$, based on the adaptive genetic algorithm as follows:


(11) $$P_{c}^{\prime }=\left\{ \begin{array}{c} \frac{0.3}{f_{m\,i\,n}-f_{m\,a\,x}}\,f^{\prime }-\frac{0.3}{f_{m\,i\,n}-f_{m\,a\,x}}\,f_{m\,a\,x}+0.6,f^{\prime }\geq f_{a\,v\,g}\\ k_{1},f^{\prime }<f_{a\,v\,g} \end{array}\right.$$

where *f*′ is the average fitness of individuals to be crossed; *f*_*min*_ is the minimum fitness; *f*_*max*_ is the maximum fitness; *f*_*avg*_ is the average fitness; and *k*_1_ is a constant.

The adaptation of each route in the pre-search period varies greatly, and thus we adopt the crossover operation for poorly fitted populations to improve the search efficiency at this stage. In the later stage, the overall fitness of several groups is close to the optimal value. At this point, $P_{c}^{\prime }$ will be adaptively resized with the *f*_*min* −_ *f*_*max*_ gap (positive correlation) and the crossover probability is adaptively adjusted by Equation (11) to search for an optimal solution according to the adaptation dominance degree. This aims to improve the search speed, such that individuals with a poor (high) fitness are more likely to be eliminated (retained), increasing the diversity of high-performance individuals.

(2) Optimization of mutation operator;

The search mode of the entire algorithm is determined by mutation probability *P*_*m*_, which prevents the algorithm from degenerating into a random search due to very large values. We set *P*_*m*_ between [0.001,0.1] and its values are determined as follows:


(12) $$P_{m}=\{\begin{array}{c} \frac{k_{2}(f_{max}-f)}{f_{max}-f_{min}}\\ k_{3} \end{array},\begin{array}{c} f\leq f_{avg}\\ \begin{array}{c} f<f_{avg} \end{array} \end{array}$$

where *f* is the fitness of mutating individuals; *f*_*min*_ is the minimum fitness; *f*_*max*_ is the maximum fitness; *k*_2_ and *k*_3_ are constants, with *k*_2_ in [0,0.001] and *k*_3_ 0.2.

The adaptive mutation probability *P*_*m*_ is altered as the algorithm progresses. The average adaptation in the early stage of the algorithm is small, the value of *f*_*max*_ and *f*_*avg*_ are distinct, and the initial adaptation of the mutation probability is relatively low. The average fitness increases with the overall population fitness as the iterations progress. Moreover, a decline in the difference between *f*_*max*_ and *f*_*avg*_ increases adaptive mutation probability *P*_*m*_, and the fixed mutation probability takes the larger fixed value of *P*_*m*_ *k*_3_ to effectively increase population diversity. At the later stage of the algorithm, an increase in the fitness of individuals in the population reduces the difference between *f*_*max*_ and *f*, and in order to ensure that the optimal solution is not affected, the adaptive mutation probability *P*_*m*_ is reduced accordingly.

#### Ant colony algorithm optimization

The ant colony algorithm (ACO) is a random search algorithm framework based on the probability distribution model parameterized by the solution space inspired by the foraging behavior of natural ant colonies (Chen et al., 2017). The ACO employs the following probability formula:


(13) $${\text{P}}_{\text{ij}}^{\text{k}}\,\left(\text{t}\right)=\left\{ \begin{array}{c} \frac{{\left[\tau_{\text{ij}}\,\left(\text{t}\right)\right]}^{\alpha }\,{\left[\eta_{\text{ij}}\,\left(\text{t}\right)\right]}^{\beta }}{\sum_{\text{s}\in {\text{allowed}}_{\text{k}}}{\left[\tau_{\text{ij}}\,\left(\text{t}\right)\right]}^{\alpha }\,{\left[\eta_{\text{ij}}\,\left(\text{t}\right)\right]}^{\beta }},j\in {\text{allowed}}_{\text{k}}\\ 0,\text{j}\notin {\text{allowed}}_{\text{k}} \end{array}\right.$$

where τ_*ij*_ is a pheromone on boundary (*i,j*) at moment *t*; η_*ij*_ is the heuristic transition factor from area *i* to area *j*; *tabu*_*k*_ is a taboo table used to record the work area that the ants have traveled thus far in order to prevent the ants from choosing a previously visited work area; *allowed*_*k*_ {*c*−*tabu*_*k*_} is a collection of work areas allowed to be accessed by ant *k* in the next step; α is the information heuristic factor, representing the relative importance of the route in the algorithm, namely, the influence of the amount of information on the ant route choice - the larger the value of α, the stronger the collaboration between the ants; and β is the expected heuristic factor, indicating the relative importance of visibility (Zhou et al., 2014).

The pheromone update formula released by the ants on their route is given as:


(14) $$\tau_{i\,j}=\left(1-\rho \right)\,\tau_{i\,j}+\sum_{k\,1}^{m}\Delta \,\tau_{i\,j}^{k}$$

(15) $$\Delta \,\tau_{i\,j}^{k}=\left\{ \begin{array}{c} \frac{1}{d_{i\,j}},\left(i,j\right)\in T^{k}\\ 0,\mathrm{otherw}\,\text{ise} \end{array}\right.$$

where, *ρ* ∈ (0,1) is the pheromone volatilization coefficient; and $\Delta \,\tau_{i\,j}^{k}$ is the pheromone released by the *k*th ant on the current route; *d*_*ij*_ is the distance between work area *i* and work area *j* ;

To prevent the algorithm from prematurely converging to the local optimal solution, we limit the pheromone concentration of each route to a pre-defined range (Equation 16). This can effectively prevent the amount of information on a specific route from being much larger than that of the rest of the route, thus avoiding the “endless loop” phenomenon.


(16) $$\tau_{i\,j}\,\left\{ \begin{array}{c} \tau_{i\,j},\tau_{m\,i\,n}<\tau_{i\,j}<\tau_{m\,a\,x}\\ \tau_{m\,i\,n},\tau_{i\,j}\leq \tau_{m\,i\,n}\\ \tau_{m\,a\,x},\tau_{m\,a\,x}\leq \tau_{i\,j} \end{array}\right.$$

where τ_*max*_ and τ_*min*_ are the maximum and minimum pheromone settings, respectively.

The proposed algorithm is based on the pheromone update rule of the ant colony algorithm. We first introduce the concept of iterative cycles to optimize the pheromone update rule, and subsequently optimize the pheromone update rule using the binary method. More specifically, the ants are ranked according to the time it takes for them to traverse all regions after each iteration. Only the pheromone released by the fastest 50% of ants is retained. For the optimization, the additional pheromone is used to strengthen the route, that is,


(17) $$\tau_{i\,j}\,\left(t+1\right)=\left(1-\rho \right)\,\tau_{i\,j}\,\left(t\right)+\sum_{k\,1}^{k\,\frac{m}{2}}\left(\sum_{k\,1}^{w-1}\left(w-k\right)\,\Delta \,\tau_{i\,j}^{k}\,\left(t\right)\right)+e\,\Delta \,\tau_{i\,j}^{b\,s}\,\left(t\right)$$

(18) $$\Delta \,\tau_{i\,j}^{b\,s}\,\left(t\right)=\left\{ \begin{array}{cccc} \frac{1}{L^{b\,s}},\;f\,\left(i,j\right)\,\epsilon \,T^{b\,s} & & & \\ 0,\mathrm{otherwise} & & & \end{array}\right.$$

where e is the size of the weight given to path *T^bs^* ; $\Delta \,\tau_{i\,j}^{b\,s}\,\left(t\right)$ is the increased pheromone at time *t* of the shortest route; *T^bs^* is the shortest route; and *L^bs^* is the length of *T^bs^*.

By adjusting the pheromone update method of the ant colony algorithm, the application of the global optimal solution increases. The algorithm aims to obtain an optimal solution with fewer iterations and avoid the “endless loop” phenomenon.

#### Ant colony algorithm optimization

**FIGURE 5:**
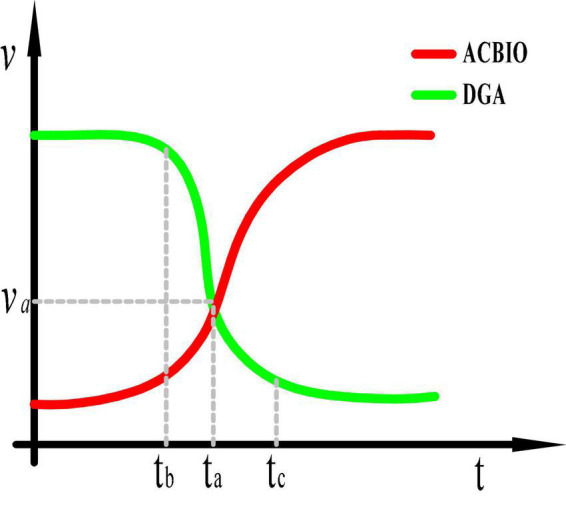
Search speeds of the dynamic genetic algorithm (DGA) and ant colony binary iterative algorithm (ACBIO).

Although the adaptive dynamic genetic algorithm optimized in this paper is able to accelerate the search efficiency, it does not overcome the low search efficiency in the later stages of the genetic algorithm. Moreover, the ant colony binary iterative algorithm is a heuristic probabilistic search method with strong local search capabilities, yet its search time is too long in the early stage. Figure 5 presents the corresponding search speed-time curves. In order to overcome the limitations of each algorithm, we integrate the optimized genetic algorithm and ant colony algorithm to produce a dynamic genetic-ant colony binary iterative fusion algorithm.

**FIGURE 6:**
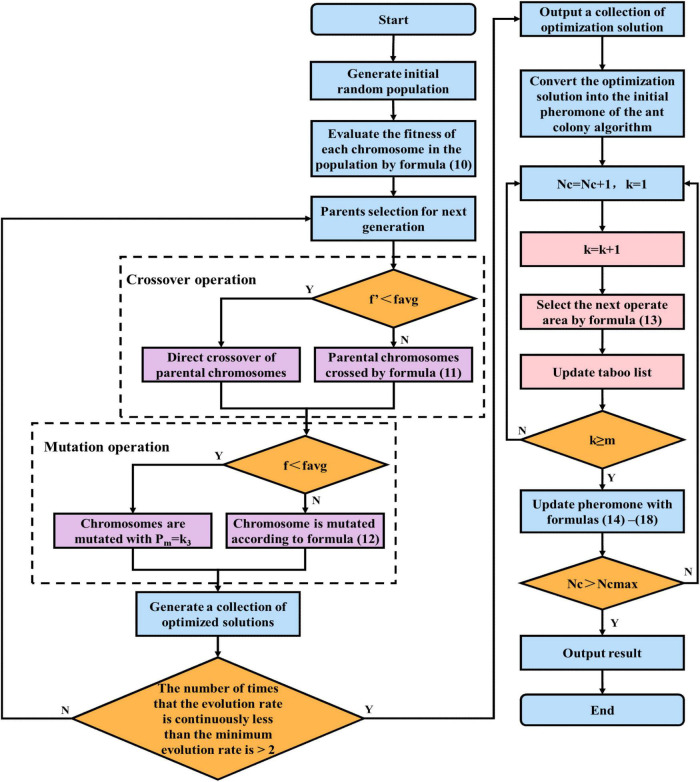
Search flow chart of DGA-ACBIO.

In this study, the fusion time is evaluated using the evolution rate of the DGA offspring population. At time tb in Figure 5, the DGA exhibits a faster search rate and evolution of individuals relative to the ACBIO, while these variables are slower after tc and are equal at ta. Therefore, the minimum evolution rate is set as the evolution rate at time ta. The evolution rate of each group iteration is subsequently compared; if the evolution rate of the population is less than the minimum evolution rate for three consecutive iterations, the DGA is terminated and the ACBIO is executed. This ensures that the algorithm obtains the optimal solution at the fastest speed. Figure 6 presents the flow chart of the proposed fusion algorithm.

### Simulation test design

#### Optimized performance tests of genetic algorithm and ant colony optimization

In order to examine the capability of the improved DGA and ACBIO in terms of search speed and accuracy, GA and ACO were evaluated by comparing the corresponding computer simulation test means with those of the optimized DGA and ACBIO, respectively. The computer used for testing had the following specifications: Intel(R) Core (TM) i7-6700HQ CPU @ 2.60 GHz and 8 GB RAM. The test was simulated on 20 tea fields using MATLAB-2018a (MathWorks) under the Win-10 platform. Table 1 reports the specific parameters of the algorithm.

**TABLE 1:** Parameters for each algorithm.

Algorithms	Parameters
GA	Population size *n* = 100, crossover probability Pc = 0.8, mutation probability Pm = 0.2, maximum number of iterations genmax = 200.
ACO	ant colony number m = 100, information heuristic factor α = 1, expected heuristic factor β = 5, maximum number of iterations genmax = 200
DGA	Population size *n* = 100, crossover probability as in Equation (2), mutation probability as in Equation (3), maximum number of iterations genmax = 200
ACBIO	ant colony number m = 100, information heuristic factor α = 1, expected heuristic factor β = 5, maximum number of iterations genmax=200
AFSA	Number of artificial fish fishnum = 100, maximum number of iterations genmax = 200, maximum number of probes trynumber = 200, sensing ranges Visual = 16, crowding factor deta = 0.8.
PSO	Evolution time nMax = 200, number of individuals indiNumber = 100, particle size parsize = 100
DGA-ACBIO	Population size *n* = 100, crossover probability as in Equation (2), mutation probability as in Equation (3), maximum number of iterations genmax = 200, genmin = 20, ant colony number = 100, information heuristic factor α = 1, expected heuristic factor β = 5

#### Dynamic genetic algorithm with ant colony binary iterative optimization search performance test

To verify the superior performance of the proposed DGA-ACBIO in terms of search speed, accuracy and number of iterations, simulations of 20 tea fields were executed and compared using the dynamic genetic algorithm, ant colony binary iterative algorithm, particle swarm algorithm (Zeng et al., 2020; Zhou et al., 2022) and artificial fish swarm algorithm (Gao et al., 2020).

For the UAV multi-tea field scheduling route planning problem, the search accuracy and performance stability of the algorithm are more important than the search time and the number of iterations. Therefore, the typical traveling salesman problems of berlin52 and kroA100 in the standard TSP LIB database, which are a close match to the multi-tea field model in this study, are selected for performance simulation tests. We verify that the DGA-ACBIO algorithm has the highest search accuracy and most stable performance with more complex targets, namely, the shortest planned flight range and the least variation in the results of each output, compared to state-of-the-art algorithms.

The maximum number of iterations and population size were set to 200 and 100, respectively. Table 1 lists the values of the other parameters, which were set according to the literature. By considering the randomness of the heuristic algorithm, each algorithm was executed 20 times.

We adopt the nonparametric Wilcoxon rank sum test set to determine significant differences between the results obtained by the DGA-ACBIO in solving the multi-tea field scheduling problem and those of the other algorithms (Kochengin et al., 2019). The significance level is set at α = 5%, indicating significant differences for *p* < 5% and vice versa. Table 5 reports the *p*-values determined from the comparison.

**TABLE 2:** Comparison of algorithm performances and optimal routes.

Algorithm	Optimal route (m)	Iteration results (times)	Running time (s)
DGA	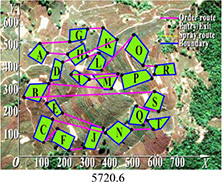	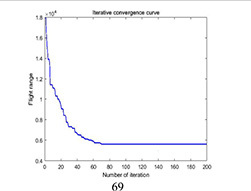	4.66
ACBIO	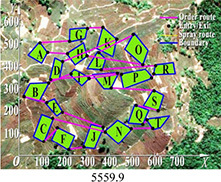	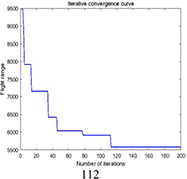	6.23
AFSA	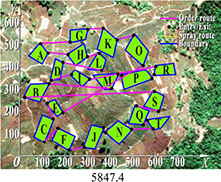	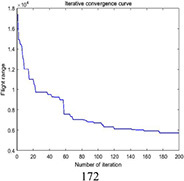	5.25
PSO	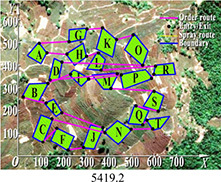	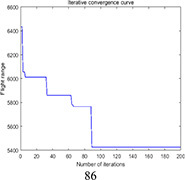	5.23
DGA-ACBIO	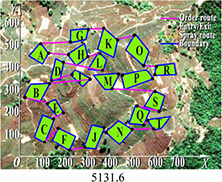	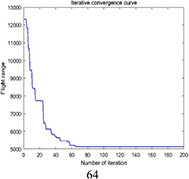	2.56

**TABLE 3:** Simulation results for different traveling salesman problems.

Problem	Algorithm	Best value (m)	Worst value (m)	Average value (m)	Range (m)	CV
Multi-tea field 20	DGA	5847.4	6571.2	6230.3	723.8	0.037
	ACBIO	5559.9	6007.9	5724.0	448.03	0.017
	AFSA	5720.6	6198.9	5904.3	478.3	0.025
	PSO	5419.2	5900.5	5566.2	481.3	0.030
	DGA-ACBIO	5131.6	5195.7	5153.4	64.1	0.005
berlin52	DGA	9450.0	11528.4	10343.8	2078.4	0.058
	ACBIO	8611.1	9326.3	8934.8	715.2	0.025
	AFSA	9245.3	10172.5	9832.5	927.2	0.026
	PSO	8819.5	9515.7	9111.9	696.2	0.022
	DGA-ACBIO	7544.4	7703.8	7603.2	159.4	0.007
kroA100	DGA	41475.1	58243.7	50356.0	16768.6	0.091
	ACBIO	33418.4	40526.3	36375.4	7107.9	0.060
	AFSA	58148.7	64499.5	61217.5	6350.8	0.028
	PSO	58127.0	64854.3	60823.0	6726.7	0.035
	DGA-ACBIO	21511.3	22198.7	21835.2	687.4	0.009

**TABLE 4:** Simulation results of each algorithm and iterative performance curve of DGA-ACBIO.

Multi-tea field20	Results of each algorithm after performing different problems 20 times	Iteration curve of DGA-ACBIO algorithm after performing different problems 20 times
	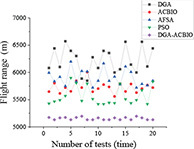	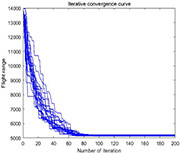
Berlin52	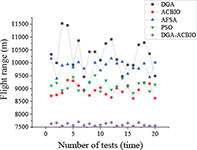	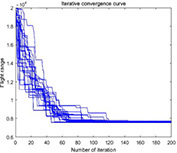
kroA100	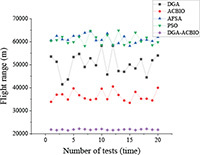	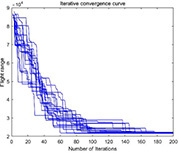

**TABLE 5:** Significance of differences in algorithm results.

Comparison of algorithms	*P*-value
DGA-ACBIO vs. DGA	1.186E-9
DGA-ACBIO vs. ACBIO	0.001540
DGA-ACBIO vs. AFSA	6.9579E-7
DGA-ACBIO vs. PSO	1.267E-7

## Results

### Genetic algorithm and ant colony optimization optimized performance tests

### Results of dynamic genetic algorithm with ant colony binary iterative optimization search performance test

(1) Table 2 compares the optimal routes and convergence curves of the DGA-ACBIO search performance test results. (2) Table 3 reports the results of each algorithm for the 20 tests on the self-built model Traveling salesman problem, as well as the standard Traveling salesman problem LIB databases berlin52 and kroA100 for the typical traveling salesman problems.

(3) Table 4 reports the results of each algorithm for the 20 test simulations on the self-built Traveling salesman problem, the standard TSP LIB database berlin52 and kroA100 problems, and the iteration curves of the DGA-ACBIO algorithm.

(3) Table 5 reports the significance levels of the differences between the DGA-ACBIO results and those of each algorithm.

### Optimized performance tests of genetic algorithm and ant colony optimization

**FIGURE 7:**
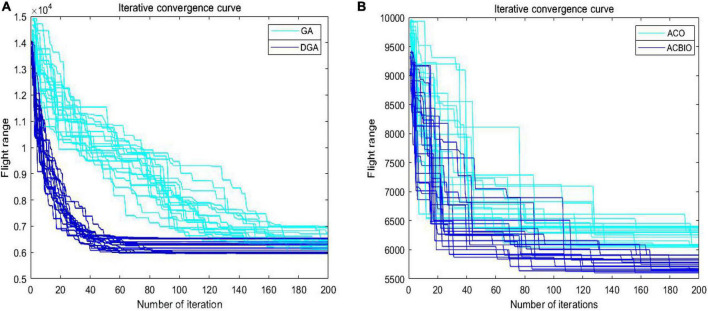
Iterative convergence curves of DGA and GA **(A)** iterative convergence curves of DGA and GA; **(B)** iterative convergence curves of ACBIO and ACO.

The iterative curves of dynamic genetic algorithm (DGA) and genetic algorithm (GA) in Figure 7A. exhibit obvious breaks in the early stage, with significant improvements in the density and smoothness of the former compared to the latter. This demonstrates the ability of DGA to directly crossover the poorly adapted individuals in the early stage and dynamically adjust the crossover and variation probability sizes according to the population adaptation dominance degree in the later stage. It can also quickly and effectively eliminate the poorly adapted individuals and improve the convergence efficiency of the algorithm in the early stage. The GA optimization approach is proved to achieve significant improvements in search speed, yet advantages in the global optimization capability are not obvious. A smaller convergence interval is observed for the ant colony binary iteration optimization (ACBIO) iteration curve compared to that of ant colony optimization (ACO), and the search accuracy and global optimization capability are significantly improved in the former (Figure 7B). This proves that the ACBIO optimization method can significantly improve the performance of the ACO search, yet advantages in search speed are not observed.

### Dynamic genetic algorithm with ant colony binary iterative optimization search performance test

#### Dynamic genetic algorithm with ant colony binary iterative optimization presents significant advantages in the optimization accuracy and speed

The optimal routes and convergence curves of the search performance simulation test results in Table 2 reveal that although the five algorithms are able to obtain flight routes traversing all tea fields, the search accuracy, speed and number of iterations vary with algorithm. The p-values of the dynamic genetic algorithm with ant colony binary iterative optimization (DGA-ACBIO) in Table 5 are all less than 5%, indicating the significantly improved optimization ability relative to the other bionic algorithms. Furthermore, the optimal solution of DGA-ACBIO exhibits the highest accuracy of 5131.6 m, while the DGA optimal solution has the worst accuracy of 5847.4 m. The optimal solution of DGA-ACBIO is 414.5 m less than the shortest result of other bionic algorithms, demonstrating the remarkable search accuracy of DGA-ACBIO.

The proposed algorithm also exhibits the shortest search time (2.56 s), while ACBIO has the longest search time (6.23 s). The search times of DGA, ACBIO, artificial fish swarm algorithm (AFSA), and particle swarm optimization (PSO) are 2.43, 1.82, 2.04, and 2.05 times that of DGA-ACBIO, respectively. Thus, DGA-ACBIO has the fastest search speed of the tested algorithms, indicating the successful optimization of the algorithm search speed performance in this study.

DGA-ACBIO presents the least number of iterations (64) and AFSA the most (172). Compared with the other bionic algorithms, DGA-ACBIO on average reduces the number of iterations by 45.75, proving its ability to obtain the optimal solution with fewer iterations.

The analysis indicates the DGA optimal solution to have the worst accuracy and ACBIO to have the longest search time. Despite this, the fusion of these two algorithms to obtain DGA-ACBIO significantly improves the search accuracy and stability, with the highest search accuracy and shortest search time among the tested algorithms.

The mean optimal solutions of DGA-ACBIO are 5153.4, 7603.2, and 21826.2 m for the self-built model Traveling salesman problem, the Berlin52 problem and the kroA100 problem solved by the LIB database, respectively. These values are much smaller than those corresponding to the other four bionic algorithms (Table 3). This is most obvious in the traveling salesman problem solution of kroA100, where the optimal solutions of DGA, ACBIO, AFSA, and PSO are 2.34, 1.69, 2.84, and 2.83 times that of the DGA-ACBIO results, respectively. This indicates the great superiority of DGA-ACBIO in the optimal solution accuracy, and the more complex the target, the more obvious the advantage. Moreover, DGA-ACBIO had the smallest range and coefficient of variation (687.4 and 0.009, respectively), demonstrating the higher credibility of the results. The DGA-ACBIO designed in this study not only has a high optimization accuracy, but also highly stable results, which proves the improved performance of the algorithm optimization.

This is attributed to the initial optimization search via DGA to rapidly obtain the optimized solution followed by the secondary search using ACBIO for the suboptimal solution, thus optimizing the solution. The first step of the DGA-ACBIO solution, namely, the implementation of the DGA, to some extent determines the optimal solution size of the algorithm. Comparing the iteration curves of the DGA and GA algorithms in Figure 7A shows that the improvement of GA in this paper can substantially enhance the efficiency of the algorithm’s preliminary search. More specifically, it can improve the evolutionary efficiency of the algorithm, optimize the overall size of the solution in fewer iterations, and reduce the search time. The iteration curves of the DGA and GA algorithms in Figure 4. reveal the improvement of GA in this paper to substantially enhance the efficiency of the algorithm’s preliminary search, namely. The evolutionary efficiency is increased, the overall size of the solution is optimized in fewer iterations, and the search time is lowered. Although the advantage is not obvious in terms of the search accuracy, an optimized solution set can be obtained in fewer iterations.

Despite its advantage in the search accuracy, it may not be possible to obtain the optimal solution by the arithmetic power of ACBIO alone within a limited maximum number of 200 iterations (Figure 7B). Since the nature of ACBIO is a positive feedback mechanism, the suboptimal solution will hold a greater advantage if it is obtained first, causing the algorithm to focus on the better candidate solution earlier. This mechanism reduces the population diversity and limits the global optimization capability of the algorithm (Chen et al., 2021; Skinderowicz, 2022). As shown in Figure 7A and the left panel of Table 4, GA does not exhibit great improvements in the global optimization capability. Taking the average solution of ACBIO as the reference of the suboptimal solution, the average solution of DGA is different by 10, 15, and 38% to the suboptimal solution, respectively. These gaps indicate that although the DGA can effectively optimize the prior solution, the optimization is not sufficient to reach the improved candidate solution of ACBIO. Moreover, the dynamic crossover and variation probabilities proposed in this study enrich the output population diversity and reserve enough optimization space for ACBIO in the fusion algorithm. Thus, ACBIO is prevented from falling into a local optimum prematurely to some extent. Although ACO is not observed to obviously improve the search speed, it can significantly improve the convergence accuracy and global optimization ability of ACO (Figure 7B). The serial fusion of the two algorithms provides the ACBIO algorithm with enough iterations (under an iteration limit of 200) to take full advantage of the high search accuracy. This not only speeds up the search speed, but also improves the search accuracy to achieve the effect that 1+1 is greater than 2.

#### Strong stability advantage of dynamic genetic algorithm with ant colony binary iterative optimization search results

The DGA-ACBIO search results do not fluctuate much (right panel of Table 4), which proves that the search performance of the algorithm is more stable. The 20-test simulation results of DGA-ACBIO and DGA in the left panel of Table 4 reveal that DGA-ACBIO overcomes the poor stability of DGA, significantly improving the search result stability. Furthermore, DGA-ACBIO exhibits less fluctuation in the output results than ACBIO when smaller solutions are obtained. The reason behind this is two-fold: (i) the final solution is a double search based on the optimized solution set, limiting the difference in the output results; and (ii) since the nature of ACBIO is to use a positive feedback mechanism, and the difference in the output results is small. Therefore, DGA-ACBIO possesses both the characteristics of high stability of the ACBIO output results and the rapid DGA pre-search.

The left panel in Table 4 shows that there are minimal differences between the simulation results of all algorithms for the solutions of the self-built model Traveling salesman problem and the berlin52 problem. However, when solving the kroA100 problem, the PSO and AFSA, which originally exhibited better output results, performed poorly, with relatively large differences compared to the DGA-ACBIO results. The mechanism of each of these algorithms and the way they are programmed are distinct, and thus may not improve in a limited number of iterations. However, DGA-ACBIO is not affected by the aforementioned problems, and as shown in Tables 4, 5 the differences between DGA-ACBIO and DGA, ACBIO, AFSA, and PSO are significant. This proves that DGA-ACBIO can effectively solve the kroA100 problem within 200 generations, indicating its wider adaptability and strong performance in self-modeling multi-tea fields problems, as well as its ability to quickly obtain the optimal solution of more complex Traveling salesman problem.

## Conclusion

In this study, the optimal scheduling route planning problem for UAV operations over multiple tea fields is transformed into a Traveling salesman problem by modeling the multi-tea field environment. The adaptive dynamic genetic algorithm (DGA) is proposed by improving the crossover and variation operators of the genetic algorithm (GA). These operators are dynamically adjusted in real time with individual fitness values to quickly and effectively eliminate poorly adapted individuals and to improve the search efficiency at the early stage of the algorithm. The ant colony binary iterative algorithm (ACBIO) is proposed to improve the search speed and accuracy of the algorithm by introducing the iteration cycle and acting as reinforcement to the pheromone update rule of the ant colony algorithm (ACO). The optimized algorithm is serially fused to obtain the dynamic genetic algorithm with ant colony binary iterative optimization (DGA-ACBIO), optimizing the UAV operation route for activities including the application of fertilizer in multi-tea fields.

The optimization performance tests of GA and ACO reveal the capability of the GA optimization method to significantly improve the search speed, yet advantages in the search accuracy are not clear. The ACBIO optimization approach can significantly improve the search accuracy and performance of ACO, while this is not true for the search speed. Comparisons of the optimization performance of various bionic algorithms prove the proposed DGA-ACBIO to have a significant superiority in the optimization accuracy, speed, number of iterations and adaptability. As the number and complexity of the objectives increase, the superiority of the DGA-ACBIO algorithm performance becomes more obvious, and it can solve more complex Traveling salesman problems in a timely manner. The algorithm can streamline the route and improve the efficiency of plant protection. This research can provide technical support for the multi-area scheduling route planning of multi-tea field UAVs and manned helicopters in hilly mountainous areas, as well as basic theoretical support for the research of aerial precision operation technology, and can also act as a reference for the investigation of the Traveling salesman problem.

Future research on the global task scheduling planning of UAV plant protection will face more challenges. For example, the introduction of more constraints, such as recharging and mission time constraints (Pinto et al., 2020; Lin et al., 2022), or dynamic environmental constraints (Zhang et al., 2020), making the classical optimization algorithm challenging to solve. However, the aforementioned studies typically focus on finding the optimal safe path to a set destination, which differs from the plant protection approach in this paper. The outstanding performance of the proposed DGA-ACBIO in this complex optimization problem is attributed to its excellent global optimization capability and the ability to avoid local minima. Nevertheless, due to the limitation of the battery range of the plant protection UAV, this study did not conduct simulation experiments over a wide range of tea fields. Future work will explore UAV scheduling route planning for multiple tea fields in hilly mountainous regions based on optimal energy recharge areas.

## Data availability statement

The original contributions presented in this study are included in the article/Supplementary material, further inquiries can be directed to the corresponding author/s.

## Author contributions

PZ and YR collected, analyzed the data, and wrote the manuscript. DW and FM collected and analyzed the data. SW and NY wrote the manuscript. ZL conceived the project. YL conceived, designed, supervised the experiments, analyzed the data, and wrote the manuscript. All authors reviewed the manuscript.
